# Effects of Recombinant Human Thyrotropin Administration on 24-Hour Arterial Pressure in Female Undergoing Evaluation for Differentiated Thyroid Cancer

**DOI:** 10.1155/2014/270213

**Published:** 2014-08-18

**Authors:** Gianna Rentziou, Katerina Saltiki, Efstathios Manios, Kimon Stamatelopoulos, Eleni Koroboki, Anastasia Vemmou, Emily Mantzou, Nikolaos Zakopoulos, Maria Alevizaki

**Affiliations:** ^1^Endocrine Unit, Department Medical Therapeutics, Alexandra Hospital, Athens University School of Medicine, 80 Vass Sofias Avenue, 11528 Athens, Greece; ^2^Endocrine Unit, Evgenidion Hospital, Athens University School of Medicine, 80 Vass Sofias Avenue, Athens, Greece; ^3^Hypertension Unit, Department of Medical Therapeutics, Alexandra Hospital, Athens University School of Medicine, 80 Vass Sofias Avenue, 11528 Athens, Greece; ^4^Vascular Laboratory, Department of Medical Therapeutics, Alexandra Hospital, Athens University School of Medicine, 80 Vass Sofias Avenue, 11528 Athens, Greece

## Abstract

*Objective.* Thyroid-stimulating-hormone (TSH) receptors are expressed in endothelial cells. We investigated whether elevated TSH levels after acute recombinant TSH (rhTSH) administration may result in alterations in blood pressure (BP) in premenopausal women with well-differentiated thyroid carcinoma (DTC).* Designs.* Thirty euthyroid DTC female patients were evaluated by rhTSH stimulation test (mean age 40.4 ± 8.6 years). A 24 h ambulatory systolic and diastolic blood pressure (SBP, DBP) monitoring (24 hr ABPM) was performed on days 2-3(D2-3). TSH was measured on day 1(D1), day 3(D3), and day 5(D5). Central blood pressure was evaluated on D3. Twenty-three patients were studied 1–4 weeks earlier (basal measurements).* Results.* TSH levels were D1: median 0.2 mU/L, D3: median 115.0 mU/L, and D5: median 14.6 mU/L. There were no significant associations between TSH on D1 and D3 and any BP measurements. Median D5 office-SBP and 24 h SBP, DBP, and central SBP were correlated with D5-TSH (*P* < 0.04). In those where a basal 24 h ABPM had been performed median pulse pressure was higher after rhTSH-test (*P* = 0.02).* Conclusions.* TSH, when acutely elevated, may slightly increase SBP, DBP, and central SBP. This agrees with previous reports showing positive associations of BP with TSH.

## 1. Introduction

It is well known that thyroid hormones influence cardiovascular function and may modulate the vascular response, the endothelial function, and the arterial blood pressure [1]. Moreover, many studies have correlated subclinical hypothyroidism with atherosclerosis, endothelial dysfunction, and coronary heart disease [2, 3]. Recently, it has been shown that thyroid stimulating hormone (TSH) levels even within the normal range are positively associated with systolic and diastolic blood pressure [4, 5]. This may suggest a direct action of TSH on the cardiovascular system especially as extrathyroidal actions of TSH have been reported [6].

TSH receptors have indeed been recognized in many extrathyroidal tissues such as smooth muscle cells and adipose tissue including endothelial cells [6–8]. Furthermore, in vitro studies have shown that TSH may alter the production of various vascular factors [9]. The question of whether TSH* per se*, after binding to receptors expressed in the vasculature, may modulate its function and if such effect could be of any clinical importanc, has not been thoroughly investigated.

TSH has a stimulatory effect on both residual normal thyroid and malignant tissue in patients with well differentiated thyroid carcinoma. Suppression of endogenous TSH levels with thyroxine is the cornerstone of therapy in differentiated thyroid cancer (DTC). Thyroglobulin (Tg) measurement is used to evaluate residual/recurrent disease; this is reliable only when TSH is high. The exogenous administration of recombinant human TSH (rhTSH) results in stimulation of Tg analogous to thyroxine withdrawal [10] and has the advantage that hypothyroidism is avoided. Thus, nowadays rhTSH is frequently administered to patients with DTC to evaluate cure of disease and offers the opportunity to study its effects in vivo. Several studies have investigated the effect of rhTSH on tissues like bone, adipose, and the cardiovascular system [11]. It has been reported that rhTSH may influence endothelial function as well as markers of inflammation [12]; however the results of previous studies are controversial showing either impairment [13] or enhancement [14] of endothelium-dependent vasodilatation.

Concerning the effect of acute rhTSH administration on blood pressure, only limited data exist: two studies have not found any association of TSH levels with single blood pressure measurements [15, 16]. There are no data of a more thorough investigation of such influence of rhTSH on day and night blood pressure.

Thus, the aim of our study was to investigate whether elevated TSH levels after acute rhTSH administration may result in alterations in blood pressure in a 24-hour ambulatory blood pressure monitoring (ABPM) as well as in central arterial pressure in individuals who had undergone thyroid ablation with radioactive iodine for well differentiated thyroid carcinoma and who have in the reevaluation remission of disease. In a subgroup of these patients a second ABPM and central blood pressure were measured 1–4 weeks before rhTSH administration in order to compare the rhTSH effect on blood pressure with basal measurements.

## 2. Materials and Methods

Sixty-three consecutive women visited the endocrine unit of our department from 2009 to 2011 for a scheduled visit, one year after total thyroidectomy, to undergo a rhTSH stimulation test for the evaluation of well differentiated thyroid carcinoma progress (DTC) (papillary or follicular). All patients were receiving TSH suppressive treatment with stable dose of levothyroxine and had fT4 and T3 levels within normal range. For the purposes of the study in order to exclude the influence of sex hormones in the cardiovascular parameters we included only women of reproductive age [17, 18]. Because of the effect of sex hormones on cardiovascular parameters, the study was always performed in the follicular phase of the menstrual cycle.

*Exclusion criteria* were menopausal women, history of known cardiovascular disease (CVD) (arterial hypertension, dyslipidemia, coronary disease, and arrhythmia), diabetes mellitus, and impaired glucose tolerance, as well as alcohol consumption, chronic hepatic or renal disease, and history of pulmonary diseases, vasculitis, or other autoimmune diseases. Obese patients were excluded only when they also had at least one further risk factor for CVD, as it has been shown that metabolically healthy obese subjects without risk factors for CVD appear to have the same risk for CVD as the nonobese [19]. Smokers were advised to abstain from smoking for at least 12 hrs before the respective tests and were not excluded; it has been reported that smoking may affect endothelial function; however, this influence in young healthy adults is rapidly reversible after smoking cessation [20, 21]. Patients with recurrence of DTC (detectable Tg levels in serum) during rhTSH stimulation test were also excluded from the analysis.

13 patients did not fulfill the criteria of our study (3 women had a history of hypertension and dyslipidemia, 4 had history of dyslipidemia and obesity, 3 had a history of impaired glucose tolerance or diabetes mellitus, 1 received drugs for hypertension, and 2 were menopausal). 4 patients proved to have persistent disease during rhTSH stimulation test, 14 patients denied the arterial pressure measurements and vessel examination for personal reasons, and 2 women underwent 24-hour ABPM recording but the measurement was incomplete because of technical problems and their data were not analyzed. Thus, 30 patients were finally included in the analysis.

Patients were defined as hypertensive when office systolic BP was ≥140 mmHg and/or office diastolic BP was ≥90 mmHg [22]. Diabetes mellitus was defined as fasting plasma glucose ≥126 mg/dL (fasting for at least 8 hours) and impaired glucose tolerance as fasting blood glucose levels 100–125 mg/dL (5.6–6.9 mmol/L) on two occasions during the current visit or if previously diagnosed according to the American Diabetes Association criteria [23]. Dyslipidemia, concerning patients with less than two cardiovascular risk factors, was defined as total blood cholesterol of more than 200 mg/dL or low density cholesterol (LDL) of more than 160 mg/dL according to the guidelines [24]. Overweight was defined as BMI ≥ 25 kg/m^2^ and obesity as BMI ≥ 30 kg/m^2^, divided into three categories according to BMI: Class I (30–34.9), Class II (35–39.9), and Class III (≥40 kg/m^2^) [25].

Patients participating in the study did not receive any drug for cardiovascular disease (diuretics, a- or b-blockers, ACE or renin or AT-II inhibitors, aldosterone receptor antagonists, calcium channel blocking antagonists, statins or other cholesterol lowering agents, antiarrhythmics of any category, digitalis glycosides, nitrates, anticoagulants, or antiplatelets agents). They did not receive any other drug except from thyroxine daily. They had never received chemotherapy for any other cancer type. Our patients did not have detectable antithyroglobuline antibodies (anti-Tg) and thus they did not receive radioactive iodine to perform a whole body scan during the stimulation test. Eight of our patients were smokers and 19–30 years old and they did not have other risk factors for cardiovascular disease; they were recommended to smoke a stable amount of cigarettes during measurements and they had abstained from smoking for at least twelve hours before blood pressure and vascular measurements.

We performed a 24-hour ABPM and central blood pressure assessment during rhTSH stimulation test. Twenty three patients underwent twice 24 h ABPM and central blood pressure assessment: during TSH stimulation (rhTSH: 24 h ABPM, rhTSH: central BP) as well as 1–4 weeks before the test (basal:24 h ABPM, basal: central BP). Due to personal reasons the remaining 7 women did not undergo the basal evaluation 1–4 weeks before the performance of the test.

We recorded clinical data for our patients including personal history and any drug treatment received during the last year. We also obtained weight and height measurements. The study was approved by the local Ethics Committee and was conducted according to the Helsinki Declaration. All patients gave their written informed consent.

### 2.1. Hormonal and Biochemical Investigation

All patients were examined after an overnight fast and had abstained from smoking, caffeine, and alcohol for at least 12 hours. They received two intramuscular rhTSH injections (0.9 mg/day) on two consecutive days (D1, D2). At D1 (before rhTSH administration) blood fasting morning samples were collected by venipuncture for serum determination of the following parameters: TSH, Tg, fT4, total triidothyronine (T3), fasting glucose, total cholesterol, triglycerides (TG), high-density lipoporotein (HDL), and low-density lipoprotein (LDL). At D3 and D5 TSH and Tg were also measured. Serum TSH, free thyroxine (fT4), total triidothyronine (T3), and thyroglobulin (Tg) were measured using chemiluminescent immunometric assays with the DPC Immulite 2000 (Siemens AG HealthCare Sector, Erlangen, Germany). Reference range was for TSH: 0.36–4 mU/L, for fT4: 9–25 pmol/L, and for T3 0.7–1.8 ng/mL. The coefficient intra- and interassay variability was for TSH: 3.8% and 4.5% at 19 mU/L, for fT4: 4.8% and 6% at 19.17 pmol/l, and for total T3: 7% and 10% at 0.96 ng/mL, respectively. The analytical sensitivity for Tg was 0.2 ng/mL. All samples were stored at −20°C until hormone analysis could be performed with the same batches of assays. Biochemical parameters (glucose, cholesterol, triglycerides, and HDL) obtained on D1 were measured immediately using an automated analyser Integra 400 (Roche). LDL was calculated by the equation: [total cholesterol]-[HDL]-[(TG/5)] (Friedewald formula).

### 2.2. Blood Pressure Measurements

#### 2.2.1. Office Blood Pressure

Office blood pressure measurements (systolic and diastolic arterial pressure) and heart rate (HR) were recorded during each visit on D1, D3, and D5 by means of an automated sphygmomanometer (OMRON 705IT). Blood pressure measurements were obtained twice at a sitting position, after 15 min rest. The mean was calculated.

#### 2.2.2. 24 h ABPM

The 30 women finally participating in the study underwent 24-hour ambulatory blood pressure measurements (24 h ABPM) after the second administration of rhTSH (D2-D3). As already mentioned, 23 women had undergone a further ABPM, 1–4 weeks before rhTSH administration (basal:24 h ABPM).

The 24 h ABPM was performed on a usual working day for the patients. Readings were taken every 15 minutes starting at 12:00-13:00 am, after the central blood pressure measurements. The median 24-hour, daytime and nighttime, systolic BP (SBP), and diastolic BP (DBP) were recorded with an oscillometric Spacelabs 90207 (SpaceLabs, Issaquah Washington, U.S.) device. Additionally, the standard deviation (SD) of the above mentioned BP parameters was calculated. The median 24 h, daytime and nighttime, pulse pressure (PP) was estimated as the difference between SBP and DBP. None of our patients was hospitalized during the recording. Examination was valid if at least three recordings were available during an hour.

#### 2.2.3. Central Blood Pressure Assessment

Central blood pressures (systolic and diastolic) were evaluated the day after the second administration of rhTSH (D3) in all participants as well as 1–4 weeks before the rhTSH stimulation in 23 subjects (basal: central blood pressure). The study protocol was performed in a dark, quiet examination room after a 20 min rest for the patients. All data were collected in the morning during 8:30–11 a.m.

We used radial artery tonometry to analyze the pulse waveform of the aorta (Sphygmocor System-Atcor Medical, Sydney, Australia) as has been previously described [26]. The measurement of central blood pressures may serve as a prognostic parameter when evaluating interventions that target cardiovascular disease [27]. With a hand-held high fidelity tonometer (Millar, Instruments, Houston, TX, USA) peripheral pressure waveforms were recorded at the radial artery. The calibration was performed by calculating arterial pressures measured at the brachial artery. Aortic pressure waveforms were then calculated by applying generalized transfer functions. The analysis of the derived aortic waveform allows calculation of indices that correspond mainly to measures of arterial and particularly aortic stiffness as well as to the intensity of reflected waves. Central systolic and diastolic pressures were measured from the central aortic waveform [28].

### 2.3. Statistical Analysis 

The SPSS (version 18, IBM, Armonk, New York, US) statistical package was used to perform all the statistical analysis. Descriptive data are shown as range as well as mean ± standard deviation (SD) for data normally distributed and median (Interquartile Range, IQR) when not normally distributed. The linear regression model was used for correlations between continuous variables (Pearson's correlation was used when the distribution was normal and Spearman's correlation was used when the distribution was not normal). For the comparison of the means the unpaired *t*-test was used for data not normally distributed and the Mann-Whitney rank-test for nonparametric factors. A paired *t*-test was performed to assess the differences between arterial blood pressure before and during rhTSH stimulation test in each participant in the study for parameters normally distributed otherwise Wilcoxon signed ranks test was used for nonparametric factors accordingly. A *P* value of <0.05 was taken as statistically significant. Due to the small sample size partial correlations have been performed in order to avoid the possibility of overfitting multivariate models. All nonparametric factors included in partial correlations were log transformed.

## 3. Results

The studied female subjects (*n* = 30) had a mean age 40.4 ± 8.6 years (19–51 years) and mean BMI 24.4 ± 4.0 kg/m^2^ (17.4–35.9). One patient had BMI = 35.9 kg/m^2^ with no other risk factors for CVD. We performed the analysis both including as well as excluding this patient and no differences in the results were observed. Thus, this patient was finally included. Thus 30 women underwent 24-hour ambulatory blood pressure measurements (24 h ABPM) and central blood pressure measurements during rhTSH stimulation test and of those 23 women had already undergone a further basal ABPM and basal central blood pressure measurements, 1–4 weeks before rhTSH administration.

### 3.1. Measurements during rhTSH Stimulation Test (*n* = 30)

#### 3.1.1. Hormonal Measurements

Mean fasting glucose (FFG) levels were 85.5 ± 6.7 mg/dL, total cholesterol 162.2 ± 21.9 mg/dL, triglycerides 82.4 ± 17.9 mg/dL, low density cholesterol (LDL) 86.5 ± 12.8 mg/dL and high-density cholesterol (HDL) levels 56.0 ± 9.0 mg/dL. In all subjects Tg levels remained <0.5 ng/mL before and after rhTSH stimulation test consistent with absence of residual disease. The TSH range on D1 (before the rhTSH administration) was 0–2.6 mU/L (median 0.2 mU/L, IQR 0.1), on day D3 62.0–178.1 mU/L (median 115.0 mU/L, IQR 0.4) and on D5 2.9–24.1 mU/L (median 14.6 mU/L, IQR 7.5, *P* < 0.001). All patients had fT4 and total T3 within the normal range (D1: fT4 range 16.3–2.1 pmol/l, median 19.6 pmol/l, IQR 9.1 and T3 range 0.7–1.8 ng/mL, median 1.2 ng/mL, IQR 0.3).

#### 3.1.2. Office Blood Pressure Measurements

On D1 and D3 all subjects had office arterial pressure within normal limits; however on day 5 (D5) five patients were found with blood pressure levels outside the normal limits according to the criteria mentioned above (Table 1). The office blood pressure in D1 and D3 was relatively stable (median difference for SBP: −0.6 (IQR 15) mmHg and for DBP: −0.4 (IQR 9) mmHg) while there was an increase in office blood pressure between D3 and D5 (median difference: between D5–D3 SBP: 11 (IQR 22) mmHg, between D5–D3 DBP: 7 (IQR 18) mmHg).

No significant associations between office arterial pressure measurements and the respective TSH levels on D1 and D3 were found. However, a positive correlation between day 5 office systolic arterial pressure and D5-TSH levels was observed (*r* = 0.541, *P* < 0.008, Spearman's correlation). No significant association was found between diastolic arterial pressure and D5-TSH levels (*r* = 0.340, *P* = 0.06, Spearman's correlation).

#### 3.1.3. 24 h ABPM Measurements

Concerning the 24 h ABPM measurements, no significant associations between any of the arterial pressure measurements and TSH levels on D3 were found. However, a positive correlation between Standard Deviation of SBP (SD-SBP) with D3 TSH levels was found (*r* = 0.371, *P* = 0.04, Spearman's correlation).

**Figure 1:**
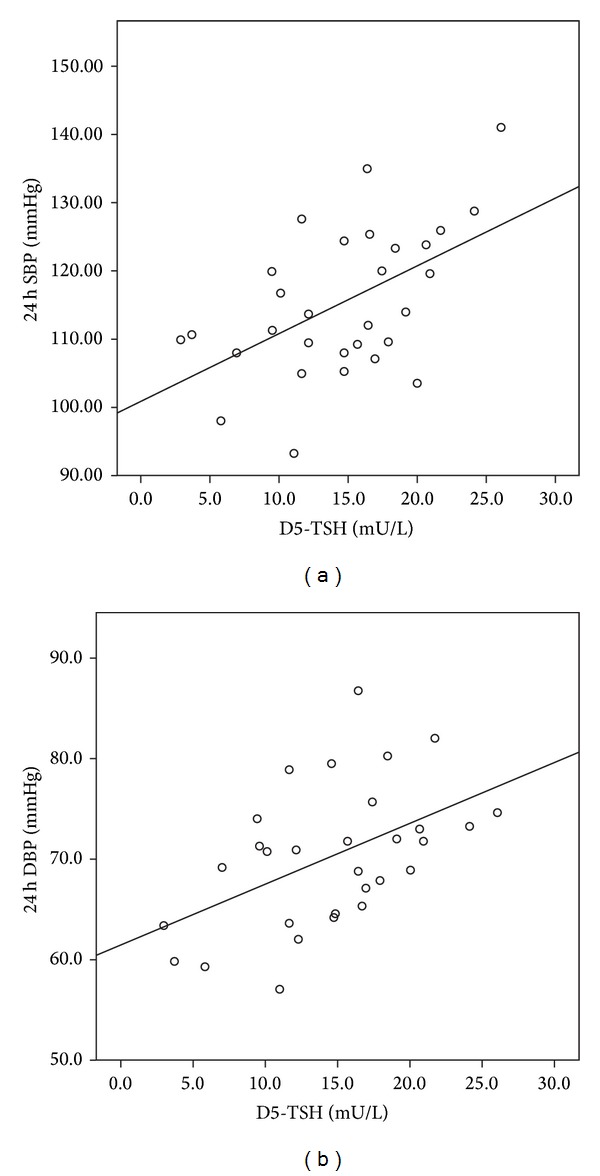
Correlation of 24 h SBP and 24 h DBP with D5-TSH levels in female patients undergoing rhTSH stimulation test.

A significant positive association of 24 h SBP and DBP with D5-TSH levels was found (for SBP: *r* = +0.442, *P* = 0.014, DBP: *r* = +0.504, *P* = 0.005, respectively, Spearman's correlation, Figure 1). Similarly significant positive correlations were observed between daytime SBP, DBP with D5-TSH levels (*r* = +0.482, *P* = 0.007, DBP: *r* = +0.548, *P* = 0.011, respectively, Spearman's correlation) as well as between nighttime SBP, DBP, and D5-TSH (SBP: *r* = +0.473, *P* = 0.008, DBP: *r* = +0.517, *P* = 0.003, Spearman's correlation).

We performed partial correlations taking into account possible confounding factors such as age, BMI, smoking, total cholesterol, and HDL levels. The association between D3-TSH levels and Standard Deviation of SBP (SD-SBP) remained significant when these confounding variables were taken into account as *r* = +0.338, *P* = 0.046, *r* = +0.430, *P* = 0.022, *r* = +0.384, *P* = 0.044, *r* = +0.370, *P* = 0.026 and *r* = +0.404, *P* = 0.035, respectively. Similarly, the association of both systolic and diastolic arterial pressure measurements with D5-TSH levels remained significant when these confounding factors were also taken into account (Table 2).

#### 3.1.4. Central Arterial Pressure Measurements

A significant correlation was found between central systolic blood pressure and D5-TSH levels (*r* = 0.503, *P* = 0.04), while no significant association was found between central diastolic blood pressure and D3 or D5 TSH levels (Table 1).

### 3.2. Comparison of Blood Pressure Measurements, Obtained during rhTSH Test, with Basal Measurements (*n* = 23)

In the subgroup of 23 patients who had undergone a basal 24 h ABPM record 1–4 weeks earlier, no significant difference in 24 h SBP and DBP was found (Table 1). Similarly, no statistically significant associations were found when daytime and nighttime measurements were analyzed separately. However the pulse pressure was significantly higher after rh-TSH administration compared to basal (Table 1). Similar differences were found when daytime and nighttime pulse pressure measurements were evaluated separately (Table 1).

Our main findings by post hoc power calculations had sufficient power. Specifically, the observed changes in 24 h PP before and after rhTSH provided 85% power (one-tailed alpha ≤0.05). In a similar manner, the observed significant correlations between BP parameters and D5-TSH also provided sufficient power ranging from 80.8% to 90% (calculated by G*power online calculator).

## 4. Discussion 

In our study we examined whether elevated TSH levels after acute rhTSH administration may result in alterations in arterial blood pressure, both peripheral and central, in DTC patients without residual disease. This is the first study in which a 24-hour monitoring of blood pressure is performed in DTC patients during a TSH stimulation test in order to examine in a more detailed manner a possible effect of acute TSH elevation on arterial pressure.

Our first important finding concerns the possible influence of rhTSH on blood pressure levels; we found that the 24 hour, daytime and nighttime, SBP, and DBP measurements in 24 h ABPM were positively associated with the increased TSH levels on day 5. We further showed that D5-TSH levels are associated with D5-office systolic blood pressure. These associations remained significant when confounding cardiovascular risk factors were taken into account in partial correlations. As the 24 h ABPM method allows the investigation of blood pressure variations even during the night, the influence of exogenous factors such as the stress of a medical procedure can be reduced or even eliminated.

Furthermore, the 24 h standard deviation (SD) of SBP was positively associated with TSH levels on D3, independently of the various confounding factors in partial correlations. This is of importance as it has been previously reported that increased blood pressure variability is an independent predictor for target-organ-damage and cardiovascular mortality [29, 30].

Finally, we found significant positive correlation of D5-TSH levels with central systolic arterial pressure. There are no previous reports in the literature about a possible association between acute increase of TSH levels and central arterial pressure. Central pressures have been proven to be a better marker than peripherally obtained blood pressures to estimate cardiovascular risk [31].

There are possible explanations concerning the association of D5-TSH levels with blood pressures obtained on day 3. First, TSH levels at D3, which are the highest, have little variations that do not allow us to observe any significant relationships during the statistical analysis. Moreover, it is possible that the increase in blood pressure levels recorded on D3 might be associated with the overall increase of TSH and not with the exact TSH levels measured on D3. Thus, a moderate TSH increase may have similar effect on blood pressure with a very high TSH level. TSH values on D5 might be more stabilized and thus they could reflect the longer exposure effect of TSH on blood pressure. Another speculation is that blood vessels reactivity could be more obvious after a prolonged exposure to higher TSH levels. Thus, it seems that the acute increase may have no immediate significant effect in blood pressure; the increase in office blood pressures observed on D5 could also point to the same direction.

Regarding the subgroup of 23 patients in which a basal 24-hour monitoring of blood pressure was performed, pulse pressure, a significant independent predictor of cardiovascular risk [32] was significantly higher during rhTSH stimulation test compared to basal pulse pressure 1–4 weeks before rhTSH administration.

Conflicting results concerning associations of TSH with blood pressure during rhTSH stimulation test have been rarely reported in the literature [15, 16]. It should be mentioned that these studies relied on single measurements only and the alterations in blood pressure (mild decrease in blood pressure) were asymptomatic and temporary. In vitro experiments have suggested a possible effect of TSH on vascular parameters towards the direction of our results [33–36].

Our results are in the same line with previous findings where slight increases of TSH levels even within the normal range are associated with increased blood pressure measurements in humans [37, 38]. Thus, it should be noted that thyroidectomized patients, undergoing rhTSH stimulation test and who receive stable and suppressive therapy with levothyroxine, could represent an ideal population as levels of thyroid hormones are stable before and during the study. These conditions allow us to recognize a direct effect of TSH* per se* on blood pressure in vivo. As many factors can influence blood pressure, our patients were strictly selected: they had no risk factors for cardiovascular disease and they had no residual thyroid disease which in theory, via the production of cytokines, could alter the blood pressure levels [39].

## 5. Limitations

The most important limitation of our study is the relatively small number of participating patients. However our sample was highly selected and homogeneous, as mentioned before. We included only apparently healthy women of reproductive age, while the measurements were performed in the same phase of the menstrual cycle so as to exclude any possible effect of sex hormones in vascular parameters. As the effect of TSH on blood pressure appears to be limited to a few mmHg and our sample was small, such effect may be difficult to be demonstrated.

Another limitation is the fact that not all patients underwent measurements before rhTSH administration. Thus, it is possible that we may have lost some significant findings.

## 6. Conclusion

The results of this study suggest that TSH, when acutely elevated, may have a prolonged direct effect on peripheral and central arterial pressures; however, this effect is only mild and temporary and probably with no clinical significance.

The significant differences in ambulatory pulse pressure, an accurate marker of arterial stiffness, between basal and after rhTSH administration measurements also supports the view that rhTSH when acutely elevated could temporarily affect blood pressure levels, albeit at a very mild degree.

## Figures and Tables

**Table 1:** Blood pressure parameters of DTC patients undergoing rhTSH stimulation test and basal measurements (the same subjects 1–4 weeks earlier).

	rh-TSH	Basal	P*
	*n* = 30	*n* = 23	
	Median (IQR)	Median (IQR)	
Office SBP day 1 (mmHg)	118.0 (17.0)	125.0 (16.1)	0.033
Office DBP day 1 (mmHg)	75.0 (10.1)	72.5 (11.2)	0.245
Office SBP day 3 (mmHg)	112.0 (23.0)		
Office DBP day 3 (mmHg)	68.5 (11.0)		
Office SBP day 5 (mmHg)	125.0 (20.2)		
Office DBP day 5 (mmHg)	77.5 (16.3)		
Office HR day 1 (bpm)	80.0 (16.1)	78.0 (10.0)	0.67
Office HR day 3 (bpm)	70.0 (16.1)		
Office HR day 5 (bpm)	74.0 (12.1)		
24-hour SBP (mmHg)	113.8 (15.6)	114.0 (22.5)	0.334
24-hour DBP (mmHg)	69.9 (8.3)	69.7 (11.9)	0.968
24-hour HR (bpm)	74.5 (9.8)	75.2 (9.1)	0.260
Day SBP (mmHg)	118.4 (16.2)	112.4 (21.7)	0.171
Day DBP (mmHg)	73.9 (12.4)	74.6 (10.1)	0.601
Day HR (bpm)	79.3 (10.7)	80.1 (9.8)	0.159
Night SBP (mmHg)	107.1 (13.9)	102.9 (19.5)	0.212
Night DBP (mmHg)	62.3 (7.3)	60.9 (10.0)	0.573
Night HR (bpm)	64.6 (11.8)	65.0 (6.2)	0.398
24-hour PP (mmHg)	43.6 (6.8)	43.0 (9.9)	0.027
Day PP (mmHg)	43.6 (5.4)	41.0 (8.6)	0.02
Night PP (mmHg)	44.4 (5.2)	42.0 (12.4)	0.024
Aortic SBP (mmHg)	108.0 (32.0)	98.0 (29.0)	0.23
Aortic DBP (mmHg)	77.0 (21.1)	71.0 (18.2)	0.28

∗Wilcoxon ranks test.

**Table 2:** Partial correlations of D5-TSH with arterial pressure measurements when various confounding factors were taken into account.

	Confounding factors				
	Age (yrs)	BMI (kg/m^2^)	Smoking	Total cholesterol (mg/dL)	HDL (mg/dL)
Office D5-SBP (mmHg)	*r* = +0.489	*r* = +0.442,	*r* = +0.516	*r* = +0.509	*r* = +0.486
	*P* = 0.011	*P* = 0.019	*P* = 0.005	*P* = 0.006	*P* = 0.009
Office D5-DBP (mmHg)	*r* = +0.397	*r* = +0.163	*r* = +0.238	*r* = +0.243	*r* = +0.216
	*P* = 0.045	*P* = 0.204	*P* = 0.110	*P* = 0.107	*P* = 0.135
24-hour SBP (mmHg)	*r* = +0.3	*r* = +0.421,	*r* = +0.495	*r* = +0.496	*r* = +0.472
	*P* = 0.065	*P* = 0.026	*P* = 0.007	*P* = 0.007	*P* = 0.011
24-hour DBP (mmHg)	*r* = +0.428	*r* = +0.384	*r* = +0.434,	*r* = +0.444	*r* = +0.412
	*P* = 0.029	*P* = 0.044	*P* = 0.021	*P* = 0.018	*P* = 0.029
Day SBP (mmHg)	*r* = +0.298	*r* = +0.425	*r* = 0.495	*r* = +0.496	*r* = +0.470
	*P* = 0.070	*P* = 0.024	*P* = 0.007	*P* = 0.007	*P* = 0.012
Day DBP (mmHg)	*r* = +0.404	*r* = +0.388	*r* = +0.423	*r* = +0.436	*r* = +0.405
	*P* = 0.041	*P* = 0.041	*P* = 0.025	*P* = 0.020	*P* = 0.032
Night SBP (mmHg)	*r* = +0.273	*r* = +0.444	*r* = +0.510	*r* = +0.511	*r* = +0.489
	*P* = 0.089	*P* = 0.018	*P* = 0.006	*P* = 0.005	*P* = 0.008
Night DBP (mmHg)	*r* = +0.422	*r* = +0.412	*r* = +0.464	*r* = +0.471	*r* = 0.439
	*P* = 0.032	*P* = 0.029	*P* = 0.013	*P* = 0.011	*P* = +0.019
